# Therapeutic Effect of *Schistosoma japonicum* Cystatin on Atherosclerotic Renal Damage

**DOI:** 10.3389/fcell.2021.760980

**Published:** 2021-11-25

**Authors:** Huijuan Yang, Hongqi Li, Weidong Chen, Zhijie Mei, Yuan Yuan, Xiaoli Wang, Liang Chu, Yu Xu, Yan Sun, Dingru Li, Hongyu Gao, Bin Zhan, Huihui Li, Xiaodi Yang

**Affiliations:** ^1^Department of Nephrology, First Affiliated Hospital of Bengbu Medical College, Bengbu, China; ^2^Anhui Key Laboratory of Infection and Immunity of Bengbu Medical College, Bengbu, China; ^3^Department of Gerontology, Anhui Provincial Hospital, First Affiliated Hospital of University of Science and Technology of China, Hefei, China; ^4^Department of Urology, First Affiliated Hospital of Bengbu Medical College, Bengbu, China; ^5^Basic Medical College of Bengbu Medical College, Bengbu, China; ^6^Second Affiliated Hospital of Bengbu Medical College, Bengbu, China; ^7^National School of Tropical Medicine, Baylor College of Medicine, Houston, TX, United States

**Keywords:** cystatin, *Schistosoma japonicum*, atherosclerosis, renal, immunoregulation

## Abstract

Atherosclerosis is a chronic inflammation of the arterial vessel wall driven by lipid metabolism disorders. Although helminthic infection and their derivatives have been identified to attenuate the chronic inflammatory diseases, the immunomodulatory effect of recombinant *Schistosoma japonicum* cystatin (r*Sj*-Cys) on metabolic diseases and atherosclerosis has not been reported. In this study, we investigated the therapeutic efficacy of r*Sj*-Cys on atherosclerotic renal damage and explored the related immunological mechanism. The results demonstrated that treatment with r*Sj-*Cys significantly reduced body weight gain, hyperlipidemia, and atherosclerosis induced by the high-fat diet in apoE^–/–^ mice. The treatment of r*Sj*-Cys also significantly improved kidney functions through promoting macrophage polarization from M1 to M2, therefore inhibiting M1 macrophage–induced inflammation. The possible mechanism underlying the regulatory effect of r*Sj*-Cys on reducing atherosclerosis and atherosclerotic renal damage is that r*Sj*-Cys stimulates regulatory T cell and M2 macrophage polarization that produce regulatory cytokines, such as interleukin 10 and transforming growth factor β. The therapeutic effect of r*Sj*-Cys on atherosclerotic renal damage is possibly through inhibiting the activation of TLR2/Myd88 signaling pathway. The results in this study provide evidence for the first time that *Schistosoma*-derived cystatin could be developed as a therapeutic agent to treat lipid metabolism disorder and atherosclerosis that threats million lives around the world.

## Introduction

Atherosclerosis is the chronic process of plaque buildup with deposition of fats, cholesterol, and other substances in and on artery walls, leading to the blood vessel hardened and narrowed and tissue ischemia (Li et al., 2015; Gistera and Hansson, 2017). Eventually, it causes a series of complications with life-threatening consequences, especially in the heart, brain, legs, arms, or kidneys. Atherosclerosis is the major cause of myocardial infarctions and cerebral strokes, as well as kidney failure (Geovanini and Libby, 2018). Since the hypothesis of lipid renal damage was put forward, studies have confirmed that renal damage and lipid metabolism disorders are interconnected and exacerbated by each other. Lipid metabolism disorder is the major cause of atherosclerosis and renal damage (Moorhead et al., 1982; Lu et al., 2019). The obesity, hypertension, and hyperlipidemia are the risk factors for atherosclerosis (van Rooy and Pretorius, 2014; Libby et al., 2019). The prevalence of atherosclerosis is increasing worldwide as a result of the economic development and the adoption of the Western lifestyle (Torres et al., 2015). At present, the pathogenesis of atherosclerosis is not fully understood. It is generally believed that abnormal lipid metabolism, chronic inflammation, oxidative stress, and immune disorder can lead to atherosclerosis (Förstermann et al., 2017; Kattoor et al., 2017; Raggi et al., 2018; Shah, 2019). More evidence demonstrated that atherosclerosis actually is a lipid-driven chronic inflammatory disorder within the arterial wall characterized by the chronic activation of macrophages. Both innate and adaptive immunoinflammatory mechanisms are involved (Taleb, 2016; Geovanini and Libby, 2018). When the arterial epithelia are activated by the oxidized fatty acids such as oxidized low-density lipoprotein (oxLDL), the chemokines and other adhesion molecules are released, leading to monocyte/lymphocyte recruitment and infiltration into the subendothelium (Tedgui and Mallat, 2006). The recruited inflammatory mononuclear phagocytes secreted inflammatory cytokines or matrix proteases that devastate the inflammation and tissue damage in the arterial walls (Binder et al., 2002; Tedgui and Mallat, 2006). The macrophages eventually become lipid-laden foam cells that accumulate in the plaque (Witztum and Steinberg, 1991). However, the immunoinflammatory response in atherosclerosis is modulated by the regulatory pathways in which the two anti-inflammatory cytokines, interleukin 10 (IL-10) and transforming growth factor β (TGF-β), play a critical role. IL-10 has potent anti-inflammatory properties on macrophages (Bogdan et al., 1991) and plays an active role in limiting the inflammatory response in the vessel wall (Tedgui and Mallat, 2001). TGF-β is a potent anti-inflammatory, immunosuppressive cytokine and plays a potential antiatherogenic role (Lutgens and Daemen, 2001). Disruption of TGF-β signaling in T cells exhibited markedly larger atherosclerotic lesions (Robertson et al., 2003).

Recent researches have demonstrated that helminthic and their derivatives modulate the host immune responses as a strategy to survive in the host. These immunomodulatory includes the induction of regulatory T cells (Tregs), anti-inflammatory cytokines, and alternately activated macrophages to reduce host immune response to the parasites to facilitate their survival in the host (Elliott and Weinstock, 2012; van der Vlugt et al., 2012; Wammes et al., 2014; Radovic et al., 2015). Based on this observation, the helminthic infection or helminth-derived products have been widely used to treat chronic inflammation–associated diseases including inflammatory bowel disease (Weinstock et al., 2005; Smith et al., 2007), asthma (Schwartz et al., 2018; Sun et al., 2019), and arthritis (Cheng et al., 2018). Because of the inflammatory property of atherosclerosis, some helminth-derived proteins have been successfully applied to alleviate atherosclerosis. Filarial nematode *Acanthocheilonema viteae*–secreted ES-82 was able to reduce lupus-associated accelerated atherosclerosis in a mouse model (Aprahamian et al., 2015). *Schistosoma mansoni* infection and its soluble egg antigens enabled to modulate macrophage inflammatory responses and protected against atherosclerosis in a mouse model (Wolfs et al., 2014). Infection of *S. mansoni* reduced 50% atherosclerotic lesions in the aortic arch associated with reduced cholesterol level in blood of mice (Doenhoff et al., 2002). *Schistosoma japonicum*–secreted cystatin (*Sj*-Cys) is a strong immunomodulatory protein to regulate macrophage activation and inhibit host inflammatory cytokines (Vray et al., 2002) and has been successfully used to treat murine collagen-induced arthritis (Liu et al., 2016), type 1 diabetes (Yan et al., 2020), trinitrobenzene sulfonic acid (TNBS)-induced experimental colitis (Wang et al., 2016), and bacterial sepsis (Li et al., 2017; Gao et al., 2020; Xie et al., 2021). Whether r*Sj*-Cys has therapeutic effect on atherosclerosis and its complications has not yet been reported. In this study, we induced atherosclerosis in apoE^–/–^ mice by feeding them with a high-fat diet (HFD) and observed the therapeutic efficacy of r*Sj*-Cys in reducing atherosclerosis and atherosclerotic renal damage.

## Materials and Methods

### Ethics Statement

Experimental animals were purchased from the animal center of Bengbu Medical College. All animal experiment protocols were reviewed and approved by the Ethics Committee of Bengbu Medical College (approval no. LAEC-2014-039).

### Production of r*Sj*-Cys

DNA-encoding *Sj*-Cys (GenBank: FJ617450) was amplified from *S. japonicum* total cDNA and cloned into yeast expression vector pPIC9k. Recombinant *Sj*-Cys (r*Sj*-Cys) with His-tag at C-terminus was expressed in *Pichia pastoris* GS115 using the method described previously (Xie et al., 2021). The contaminated endotoxin in the purified r*Sj*-Cys was removed by using a ToxOut^TM^ High Capacity Endotoxin Removal Kit (BioVision, Palo Alto, CA, United States) and confirmed by Pierce^TM^ LAL Chromogenic Endotoxin Quantitation Kit (Thermo Fisher Scientific Inc., Waltham, MA, United States). The concentration of r*Sj*-Cys was measured by BCA Protein Quantitation Kit (Beyotime Biotechnology, Shanghai, China). The purity and molecular weight were measured by sodium dodecyl sulfate–polyacrylamide gel electrophoresis (SDS-PAGE). The purified protein was stored at –80°C until use.

### Induction of Atherosclerosis in Mice and Treatments

Male apoE^–/–^ mice (with C57BL/6J background, specific pathogen free) with 7–8 weeks old were purchased from the Animal Center of Bengbu Medical College and housed in a temperature-controlled room with a 12-h light–dark cycle. All mice had *ad libitum* available for food and water. Total 12 apoE^–/–^ mice were fed with HFD (D12108C: 20% fat, 1.25% cholesterol; Xietong Biotechnology, Nanjing, China) to induce atherosclerosis (Liu et al., 2017); six of them were intraperitoneally treated with 20 μg of r*Sj*-Cys per mouse four times at the first week of HFD feeding and once a week for the following 11 weeks, whereas the other six mice received phosphate-buffered saline (PBS) only at the same regimen. For the control groups, 12 apoE^–/–^ mice were fed with normal control diet (NCD) and divided into two groups; six NCD-mice were intraperitoneally injected with r*Sj*-Cys, and the other six received PBS only at the same regimen as the treated groups. After 12 weeks, all mice were euthanized, and the sera, aortas, and kidneys were collected to evaluate the inflammatory cytokines and pathological changes by histochemical staining (Doenhoff et al., 2002).

### Isolation and Stimulation of Murine Peritoneal Macrophages

Peritoneal exudate cells were collected from apoE^–/–^ mice by flushing the peritoneal cavity with 5 mL ice-cold PBS containing 2% fetal bovine serum (FBS; Zhejiang Tianhang Biological Technology, Hangzhou, China). Cells were cultured on a six-well plate (4 × 10^6^ cells/well in 2 mL) at 37°C, 5% CO_2_ in RPMI 1640 medium containing 10% FBS, 1% penicillin/streptomycin (Beyotime Biotechnology) for 6 h. Non-adherent cells were washed away with PBS, and adherent cells were collected as peritoneal macrophages. The collected peritoneal macrophage cells were stimulated with oxLDL (Yiyuan Biotechnologies, Guangzhou, China) at 50 μg/mL and treated with r*Sj*-Cys (1 μg/mL), or cells without oxLDL were cultured with r*Sj*-Cys or medium only as controls. After 24 h, the secretion of inflammatory cytokines in the culture supernatants were detected by enzyme-linked immunosorbent assay (ELISA) kits [tumor necrosis factor α (TNF-α), IL-6, IL-10 using corresponding kits from Dakewe Biotech, Beijing, China; inducible nitric oxide synthase (iNOS), Arg-1 detection kits from Elabscience Biotechnology, Wuhan, China; and TGF-β kit from ABclonal Biotechnology, Wuhan, China].

### Blood Lipid Detection and Renal Function Assay

After the mice were fasted for 8 h, sera were isolated from blood samples of mice and stored at –80°C until use. The total cholesterol (TC) and triglyceride (TG), low-density lipoprotein cholesterol (LDL-c), high-density lipoprotein cholesterol (HDL-c), creatinine (Cr), and blood urea nitrogen (BUN) were measured by an automatic biochemical analyzer (Beckman Coulter, Brea, CA, United States).

### Detection of Oxidative Stress in Kidney and Urine Protein

The levels of oxidative stress in kidney were quantified by measuring malondialdehyde (MDA) using the MDA activity assay kit; MDA is one of the final products of polyunsaturated fatty acid peroxidation. The free radicals cause overproduction of MDA; therefore, the level of MDA is used as a marker of oxidative stress and lipid peroxidation (Gaweł et al., 2004). Superoxide dismutase (SOD) activity was measured using an SOD activity assay kit (Beyotime Biotechnology). In brief, renal tissues were homogenized using a homogenizer (Jingxin Experimental Technology, Shanghai, China); the homogenate supernatant was collected by centrifuging at 12,000*g* for 15 min at 4°C, which was used for measuring MDA at an absorbance 530–540 nm and SOD at 560 nm. The levels of MDA and SOD were standardization to unit weight of total protein content.

On the last day of the 12th week of the experiment, the urine was collected by using metabolic cage and centrifuged at 4,000 revolutions/min for 15 min at 4°C to obtain urine supernatant. Coomassie brilliant blue assay kit (Jiancheng Bioengineering Institute, Nanjing, China) was used to quantify the levels of urine albumin.

### Renal and Aortic Histological Analysis and Renal Immunohistochemical Staining

The kidney was collected from each mouse of different experimental groups and fixed with 4% paraformaldehyde. The renal tissue sections were stained with hematoxylin and eosin (HE) and neutral lipids and adipocytes stained with the oil red O staining solution (Servicebio Technology, Wuhan, China). All stained sections were examined at 200× magnification under a microscope (Nikon, Tokyo, Japan).

Heart tissues and the whole aorta were collected from each mouse of different experimental groups. The adipose tissue around the blood vessels was removed as much as possible. The aorta vessel was opened vertically and stained with the oil red O staining solution. The mouse hearts were dissected in order to obtain the aortic sinus stained with the oil red O. The aortic plaque lesion was observed and quantified.

To investigate the phenotypes of macrophages infiltrated in the kidneys, we performed immunohistochemical staining on paraffin sections of kidneys. Briefly, after being dewaxed and blocked with goat serum, the kidney tissue sections were incubated with rabbit anti-CD86 monoclonal antibody (1:300) and rabbit anti-CD206 polyclonal antibody (1:800) (Abcam, Cambridge, MA, United States) overnight at 4°C in a humidified box, followed by the horseradish peroxidase (HRP)–labeled anti-rabbit secondary antibody at room temperature for 50 min, and visualized with 3,3′-diaminobenzidine (ZSbio, Beijing, China). The nuclei were counterstained with DAPI. The sections were dehydrated and examined under microscope with 400 × magnification. Image Pro Plus 6.0 (IPP6.0) software was used for semiquantitative analysis, and the mean density was used to reflect the expression level of CD86 and CD206.

### Hemodynamic Analysis

In brief, the experimental mice were anesthetized by inhalation of isoflurane, and the hemodynamic analysis was performed by a high-resolution ultrasound imaging system (Vevo 2100; VisualSonics, Canada). The peak velocity and mean gradient of ascending aorta were measured by color Doppler echocardiography. All measurements were performed for three times at the consecutive cardiac cycles.

### Cytokine Profile in Sera and Renal Tissues

The levels of proinflammatory factor (TNF-α, IL-6, iNOS) and immunoregulatory factors (IL-10, TGF-β, Arg-1) were measured in sera of mice using corresponding ELISA kit as mentioned previously. The mRNA expression levels of these cytokines in the renal tissue were analyzed by quantitative reverse transcription–polymerase chain reaction (qRT-PCR) using primers listed in Table 1. Briefly, the total RNAs were isolated from the renal tissues using Trizol reagent (Ambion, Austin, TX, United States), and the cDNAs were synthesized from 2 μg of total RNA by using a reverse transcription kit (Thermo Fisher Scientific Inc.). Total 2 μL cDNA was used as the template in volume of 20 μL for qRT-PCR using the SYBR Green Super Mix Kit (Takara Bio Inc., Tokyo, Japan) and performed in a Roche LightCycler^®^ 96 real-time PCR system (Roche Molecular Systems, Inc., United States). The relative mRNA expression levels of each cytokine in the renal tissue were measured with the comparative △Cq method using the formula 2^–△△^^Cq^ normalized to GAPDH.

**TABLE 1:** Primer sequences used for qRT-PCR analysis.

**Gene**	**Forward primers (5′-3′)**	**Reverse primers (5′-3′)**
TNF-α	AACCTCCTCTCTGCCGTCAA	AAAGTAGACCTGCCCGGACTC
IL-6 iNOS	TGGAGTCACAGAAGGAGTGGCTAA CAAGCACCTTGGAAGAGGAG	TCTGACCACAGTGAGGAATGTCCA AAGGCCAAACACAGCATACC
IL-10	CCAAGCCTTATCGGAAATGA	TTTTCACAGGGGAGAAATCG
TGF-β	CTACAATGAGCTGCGTGTG	TGGGGTGTTGAAGGTCTC
Arg-1	CTCCAAGCCAAAGTCCTTAGAG	AGGAGCTGTCATTAGGGACATC
GAPDH	GGTTGTCTCCTGCGACTTCA	TGGTCCAGGGTTTCTTACTCC

### Detection of TLR2 and Myd88 in the Renal Tissue by Western Blot

The levels of TLR2 and Myd88 in the renal tissues were measured by Western blot with specific antibodies. Briefly, the total proteins were extracted from the renal tissues, and the concentration was measured by BCA Protein Quantitation Kit. Equal amounts of proteins were separated in 12% SDS-PAGE gel and transferred onto 0.45 μm polyvinylidene fluoride membranes (Macklin Biochemical Co., Shanghai, China). The membranes were blocked with 5% skimmed milk at room temperature for 2 h and then incubated with rabbit anti-TLR2 polyclonal antibody (1:1,500) (Abcam), or rabbit anti-Myd88 monoclonal antibody (1:1,000) (Affinity Biosciences, Cincinnati, OH, United States), or rabbit anti–β-actin polyclonal antibody (1:2,000) (Cell Signaling Technology, Danvers, MA, United States) overnight at 4°C followed by incubation with HRP-conjugated goat anti-rabbit immunoglobulin G (1:5,000) (Biosharp, Hefei, China). The recognized bands were semiquantitatively analyzed by Image Lab System. The results are expressed as ratios of TLR2 and Myd88 to β-actin control.

### Flow Cytometry Analysis of CD3^+^CD4^+^CD25^+^FoxP3^+^ Tregs in the Splenocytes

The splenocytes were isolated from spleens of experimental mice and cultured in RPMI 1640 medium (Thermo Fisher Scientific Inc.). The cell surfaces were blocked with rat anti-mouse CD16/32 antibody for 15 min at 4°C and then incubated with anti-mouse CD3ε-Pecy7, anti-mouse CD4-FITC, and anti-mouse CD25-APC (Biolegend, United States) for 30 min at 4°C in the dark. After being washed and resuspended in 300 μL fixation buffer (Thermo Fisher Scientific Inc.) for 30 min at 4°C and then in 1 mL permeabilization buffer (Thermo Fisher Scientific Inc.) for 30 min at 4°C, the cells were stained with anti-mouse Foxp3-PE (Biolegend) for 30 min at 4°C in the dark. The isotype-matched immunoglobulins (Biolegend, London, United Kingdom) and fluorescence minus one were used as controls for non-specific staining as baseline. The cells were washed three times and resuspended in 400 μL of 2% paraformaldehyde and detected by DxP Athena^TM^ flow cytometer (CYTEK, United States). All the data were analyzed using FlowJo-V10 software (BD Biosciences, United States).

### Statistical Analysis

All data are presented as the mean ± SEM (standard error of the mean), and the statistical analyses were performed using GraphPad Prism 5.0 software (GraphPad Inc., La Jolla, CA, United States). The same parameters in multigroup were compared by using one-way analysis of variance followed by the Student–Newman–Keuls test. *p* < 0.05 was regarded as statistically significant.

## Results

### Expression, Purification, and Identification of r*Sj*-Cys

**FIGURE 1:**
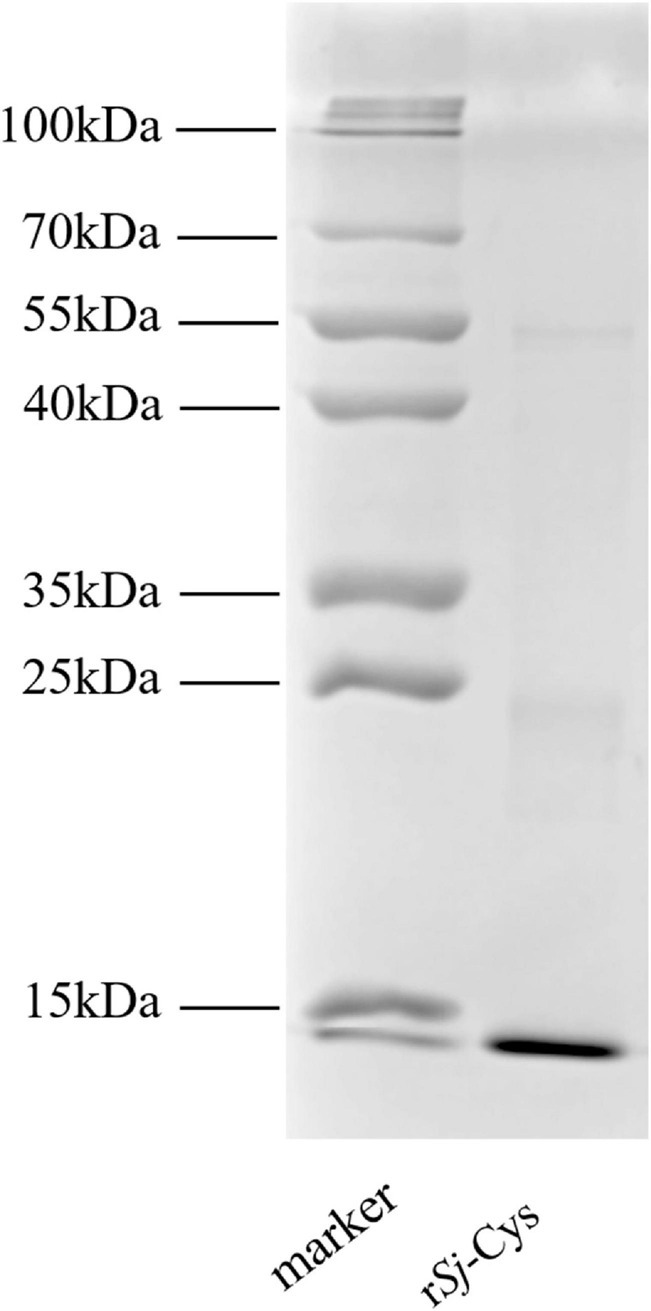
SDS-PAGE of r*Sj*-Cys. Two micrograms of purified r*Sj*-Cys was separated in 12% polyacrylamide gel.

The *Sj*-Cys protein was successfully expressed as 12-kDa soluble recombinant protein (r*Sj*-Cys) in *P. pastoris* GS115 under induction of 0.5% methanol. The r*Sj*-Cys with His-tag expressed at the C-terminus was purified using immobilized metal ion affinity chromatography and verified using 12% SDS-PAGE analysis (Figure 1). The endotoxin level kept low (0.06 EU/mL) in the protein.

### r*Sj*-Cys Treatment Reduces High-Fat Diet-Induced Body/Kidney Weight Gain in Mice

**FIGURE 2:**
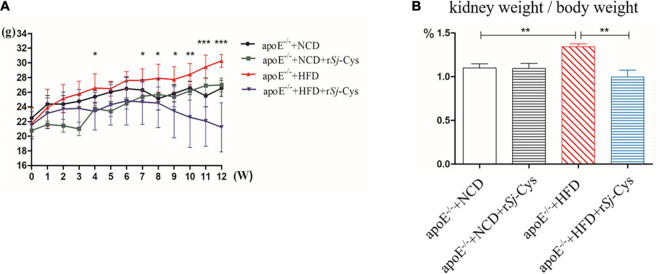
r*Sj*-Cys treatment reduced HFD-induced body weight gain in mice. **(A)** The changes in body weight throughout the experimental period. **(B)** The changes in the percentage of kidney weight/body weight ratio. The results are shown as the mean ± SEM for each group (*n* = 6 per group). *P < 0.05, **P < 0.01, ***P < 0.001.

Four weeks after being treated with 20 μg of r*Sj*-Cys, the body weight of each mouse fed with HFD started to reduce, and the body weight loss became more significant 11–12 weeks after treatment compared to the HFD mice without r*Sj*-Cys treatment (*p* < 0.01) (Figure 2A). The kidney weight–body weight ratio of HFD-fed mice treated with r*Sj*-Cys was significantly lower than those without r*Sj*-Cys treatment (Figure 2B), indicating that treatment with r*Sj*-Cys not only reduces the body weight gain in mice fed with FHD, but also even more significantly reduces kidney weight. r*Sj*-Cys itself has no effect on the body weight in mice fed with NCD.

### r*Sj*-Cys Reduces the Development of Atherosclerosis and the Histological Damage in Kidney

**FIGURE 3:**
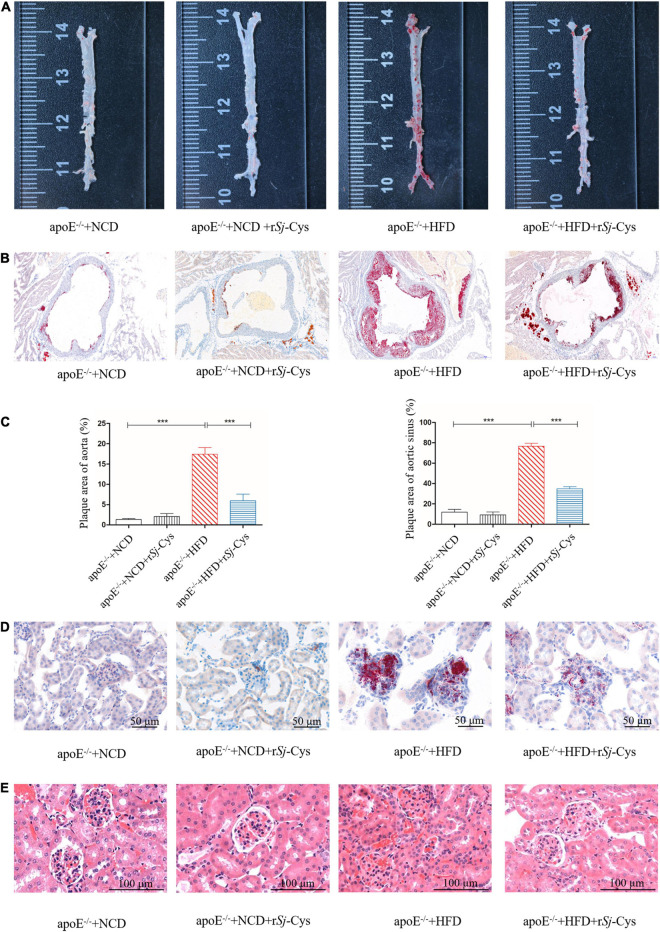
r*Sj*-Cys reduced the development of atherosclerosis. **(A)** Representative oil red O staining of aorta. **(B)** Representative oil red O staining of the aortic sinus. **(C)** The plaque area quantified as a percentage of the whole area. **(D)** r*Sj*-Cys treatment reduced glomerular fat deposition (×200, scale bar 50 μm). **(E)** Representative HE staining of the renal tissue sections (×200, scale bar 100 μm). The data are shown as the mean ± SEM for each group (*n* = 6 per group). ***P < 0.001.

We measured the development of atherosclerotic plaques on the entire aorta and aortic sinus and quantified the plaque area. The results showed that HFD feeding caused significant atherosclerotic lesions in both aorta and aortic sinus. Treatment with r*Sj*-Cys significantly reduced the atherosclerotic plaques in both aorta and aortic sinus (Figures 3A–C). Kidney histochemical examination demonstrated that fat deposition was obvious in the glomerular structure of mice fed with HFD when stained with oil red O solution (Figure 3D). HE staining showed that the glomerular structure was damaged with inflammatory cell infiltration within renal interstitium in kidneys of mice fed with HFD for 12 weeks (Figure 3E). Treatment with r*Sj*-Cys significantly reduced the fat deposition in kidney and glomerular damage caused by the HFD. There was no obvious oil red O–colored atherosclerotic lesions and renal tissue damage in groups of mice fed with NCD and NCD/r*Sj*-Cys.

### r*Sj*-Cys Treatment Alleviates Hemodynamic Disorder in Heart of Mice Fed With High-Fat Diet

**FIGURE 4:**
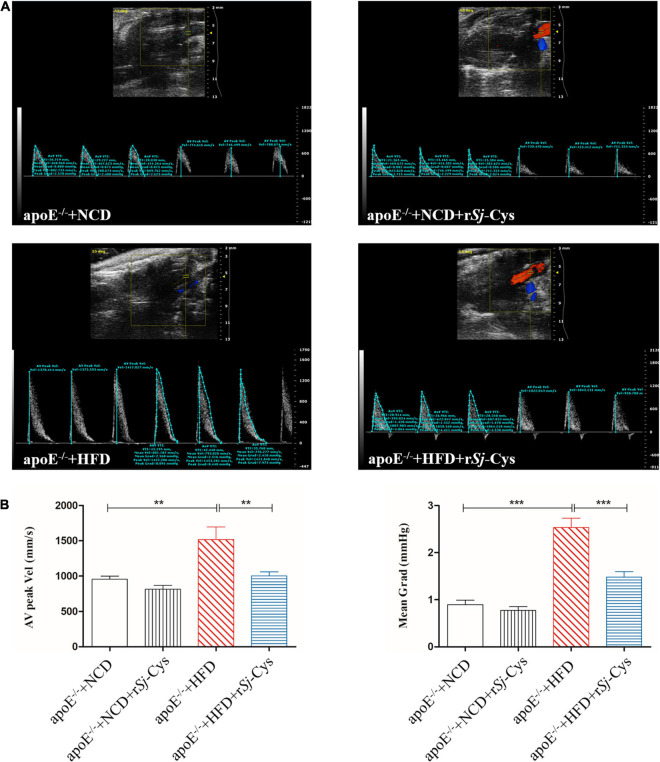
Treatment of r*Sj*-Cys improved hemodynamic function. **(A)** Representative color Doppler ultrasound of the heart. **(B)** r*Sj*-Cys improves the AV peak velocity and mean gradient of the ascending aorta. The data are shown as the mean ± SEM for each group (*n* = 6 per group). **P < 0.01, ***P < 0.001.

The blood flow of the heart was checked by echocardiography in mice 12 weeks after the treatment. As shown in Figure 4A, the peak velocity and mean gradient of ascending aorta in HFD mice were significantly increased compared to the NCD group. r*Sj*-Cys treatment significantly reversed the hemodynamic disturbances caused by HFD (Figure 4B). There was no significant change in the NCD group or group with NCD/r*Sj*-Cys.

### r*Sj*-Cys Reduces Lipid Level and Improves Renal Function of Mice Fed With High-Fat Diet

**FIGURE 5:**
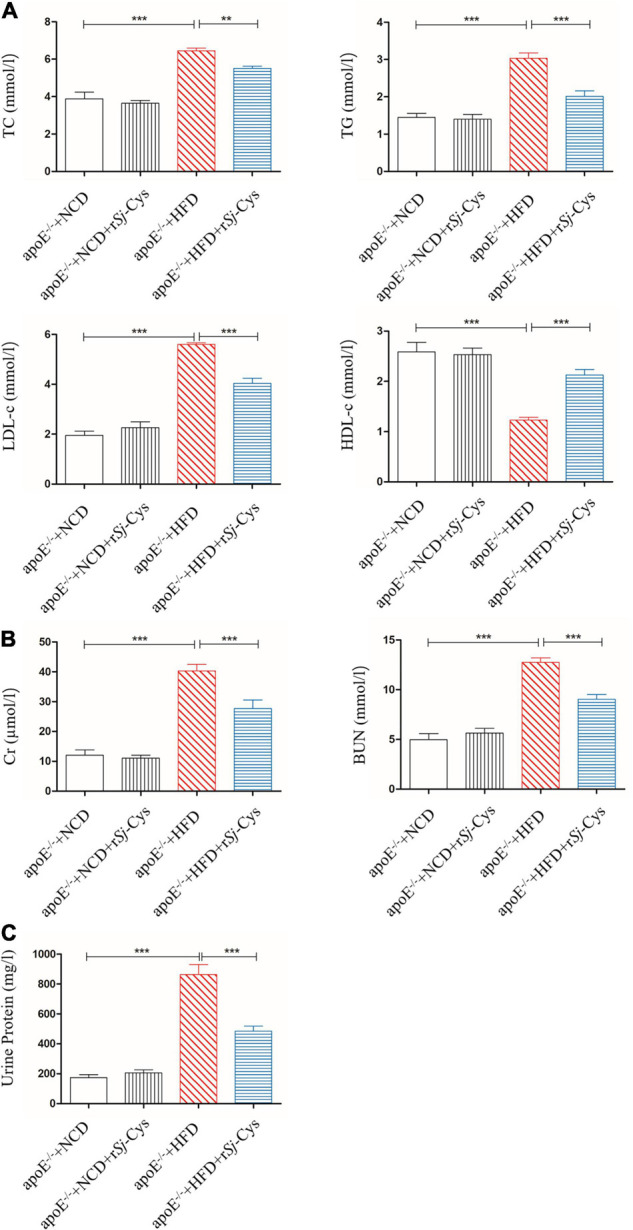
r*Sj*-Cys reduced the serum lipid levels and improved renal function. **(A)** The changes of TC, TG, LDL-c, and HDL-c in sera. **(B)** r*Sj*-Cys reduced the levels of Cr and BUN in sera. **(C)** r*Sj*-Cys reduced the levels of urine protein. The data are shown as the mean ± SEM for each group (*n* = 6 per group). **P < 0.01, ***P < 0.001.

As expected, the serum levels of TC, TG, and LDL-c were greatly increased, and the HDL-c level reduced in apoE^–/–^ mice fed with HFD compared to the mice fed with NCD. After being treated with r*Sj*-Cys, the levels of TC, TG, and LDL-c in sera were significantly reduced, and the levels of HDL-c were increased compared to the HFD-fed mice without r*Sj*-Cys treatment (Figure 5A). Feeding with HFD for 12 weeks significantly increased the level of Cr and BUN in sera and the total protein in urine, indicating the renal function was impaired after being fed with HFD; however, treatment with r*Sj*-Cys significantly reduced the levels of Cr and BUN in the sera and the protein level in the urine (Figures 5B,C), indicating that r*Sj*-Cys can regulate lipid metabolism disorders and improve kidney harmed functions.

### r*Sj*-Cys Treatment Suppresses High-Fat Diet-Induced Peroxidation in the Kidney

**FIGURE 6:**
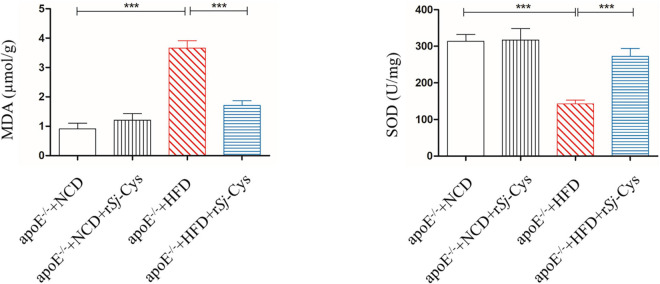
The changes of MDA and SOD in the renal tissue after the treatment of r*Sj*-Cys. The data are shown as the mean ± SEM for each group (*n* = 6 per group). ***P < 0.001.

MDA reflects the degree of lipid peroxidation and SOD as an antioxidant enzyme that protect cells from toxic oxygen metabolites. Feeding with HFD greatly increased the levels of MDA and reduced the level of SOD in kidneys of mice compared to the control mice fed with NCD; however, treatment with r*Sj*-Cys significantly reduced the MDA level and increased SOD level in kidney tissue. There were no significant differences between the NCD group and the NCD treated with r*Sj*-Cys group (Figure 6). The results indicate that treatment with r*Sj*-Cys can reduce MDA levels and increase SOD levels in kidneys of mice fed with HFD, thereby protecting the kidney from lipid peroxidation–caused damage.

### Treatment With r*Sj*-Cys Reduces M1 Macrophages and Includes M2 Macrophages in Renal Tissue of Mice Fed With High-Fat Diet

**FIGURE 7:**
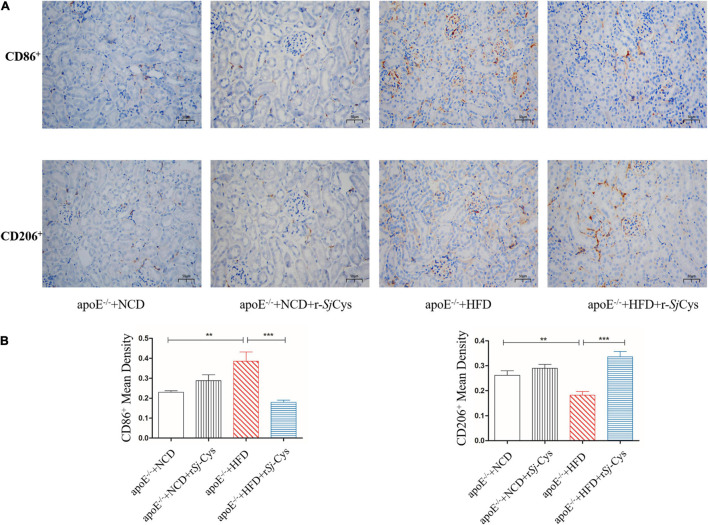
**(A)** Representative immunohistochemistry staining of CD86^+^ and CD206^+^ expression in sections of the kidney from different groups of mice (×200, scale bar 50 μm). **(B)** r*Sj*-Cys reduced the mean density of CD86^+^ and increased the mean density of CD206^+^. The results are shown as the mean ± SEM for each group (*n* = 6 per group). **P < 0.01, ***P < 0.001.

As we know, macrophage and its polarization to different phenotypes play an important role in atherosclerotic inflammation and damages (Barrett, 2020). M1 or classically activated macrophages maintain local inflammatory responses, and M2 or alternatively activated macrophages have anti-inflammatory and tissue repair/recovery properties (Colin et al., 2014). We chose CD86 as the marker for M1 macrophages and CD206 as the marker for M2 macrophages. We also performed immunohistochemical staining on the kidney to observe the effect of r*Sj*-Cys on inflammatory cell infiltration induced by HFD. The results showed that CD86^+^ M1macrophages in the kidney of the HFD-fed group were significantly increased, and the CD206^+^ M2 macrophages were significantly reduced compared with the NCD group (Figures 7A,B), indicating that feeding with HFD stimulates the M1 macrophage polarization and inflammation in kidney. Treatment with r*Sj*-Cys significantly reduced the expression of CD86^+^ and increased the expression of CD206^+^ macrophages in renal tissue (Figures 7A,B), indicating that r*Sj*-Cys stimulates the polarization of macrophages from M1 to M2 and thus reduces the HFD-induced inflammation. There were no significant differences between the NCD group and the NCD with r*Sj*-Cys group on the expression of CD86^+^ and CD206^+^ macrophages in renal tissue.

### r*Sj*-Cys Reduces the Oxidized Low-Density Lipoprotein-Induced Inflammatory Responses and Boosts Regulatory Response in Murine Peritoneal Macrophages *in vitro*

**FIGURE 8:**
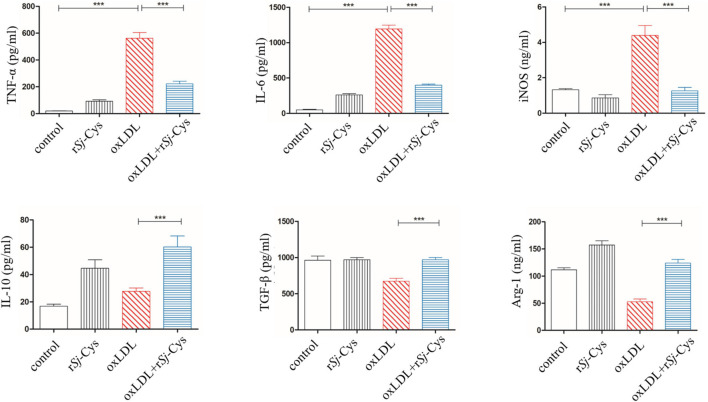
r*Sj*-Cys down-regulated proinflammatory cytokines (TNF-α, IL-6) and iNOS and up-regulated immunomodulatory cytokines (IL-10, TGF-β) and Arg-1 from murine peritoneal macrophages stimulated by oxLDL. The levels of these cytokines/enzymes in the culture supernatants were detected by ELISA 24 h after incubation. The results are shown as the mean ± SEM for each group (*n* = 6 per group). ***P < 0.001.

The oxLDL, a major risk factor for atherosclerosis, induces polarization of macrophages to the M1 phenotype and promotes inflammatory responses (Kaplan et al., 2018). In this study, we confirmed that oxLDL significantly stimulated the secretion of inflammatory cytokines TNF-α, IL-6, and iNOS in peritoneal macrophages of mice. In the presence of r*Sj*-Cys (1 μg/mL), these oxLDL-stimulated inflammatory cytokines were significantly reduced; at the same time, secretion of immunomodulatory cytokines IL-10, TGF-β, and M2 macrophage marker Arg-1 in the culture supernatant was significantly increased compared to the cells without r*Sj*-Cys treatment. The r*Sj*-Cys alone had no effect on the peritoneal macrophages (Figure 8). The results indicate that r*Sj*-Cys is able to inhibit oxLDL-stimulated inflammatory responses and increased regulatory cytokines secreted by peritoneal macrophages.

### r*Sj*-Cys Inhibits Proinflammatory Cytokines and Induces Immunoregulatory Cytokines in Mice With Atherosclerosis

**FIGURE 9:**
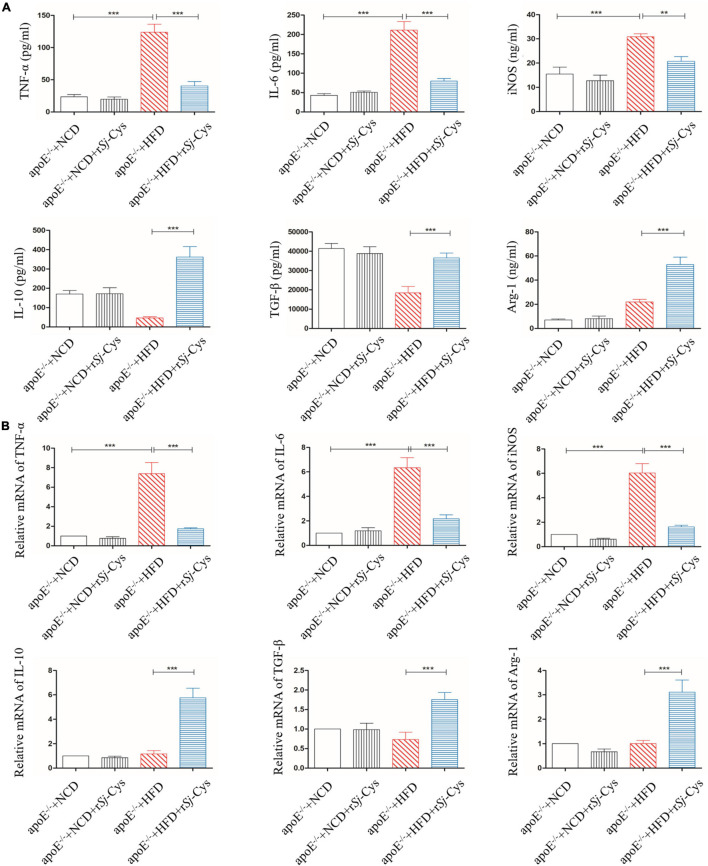
r*Sj*-Cys inhibited proinflammatory cytokines (TNF-α and IL-6) and iNOS and induced immunomodulatory cytokines (IL-10 and TGF-β) and Arg-1 in sera **(A)** and the similar mRNA expression pattern observed in renal tissues relative to the level of mice with NCD **(B)**. The data are shown as the mean ± SE for each group (*n* = 6 per group). **P < 0.01, ***P < 0.001.

In order to explore the immune mechanism underlying the renal damage caused by HFD-induced atherosclerosis, we tested the levels of proinflammatory cytokines (TNF-α, IL-6) and immunoregulatory cytokines (IL-10, TGF-β) or M1-related iNOS and M2-related Arg-1 in the sera and measured their mRNA expression levels in the kidneys. Similar to the peritoneal macrophages stimulated by oxLDL *in vitro*, the mice fed with HFD significantly increased the expression levels of TNF-α, IL-6, and iNOS in the sera (Figure 9A) and the transcriptional levels of their mRNAs in renal tissues (Figure 9B) compared to mice fed with NCD and NCD with r*Sj*-Cys. However, treatment with r*Sj*-Cys significantly reduced the expression levels of these proinflammatory cytokines and iNOS, but increased the expression levels of regulatory cytokines IL-10 and TGF-β and M2-related Arg-1 in both protein levels in sera and mRNA levels in renal tissues. There were no significant differences of them in sera of mice or their mRNA in renal tissues between the NCD group and the NCD group with r*Sj*-Cys (Figure 9B). The results indicate that treatment with r*Sj*-Cys reduces HFD-induced atherosclerosis and aortic/renal damage possibly through stimulating the polarization of M1 macrophages to M2 macrophages, therefore reducing inflammation and boosting tissue repair and recovery.

### r*Sj*-Cys Decreases the Expression of TLR2 and Myd88 in Atherosclerotic Kidney

**FIGURE 10:**
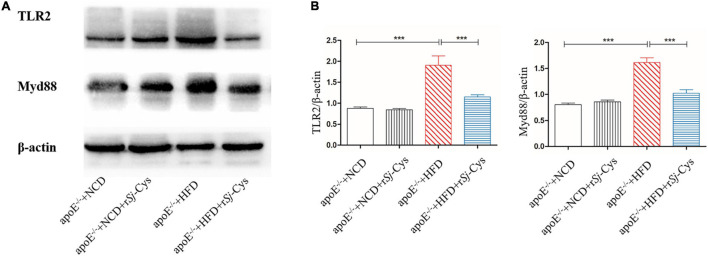
r*Sj*-Cys reduced the expression of TLR2, MyD88 in the kidneys of mice detected by Western blot **(A)**. The β-actin was measured as control. The density ratios of TLR2/β-actin and MyD88/β-actin are shown on the right **(B)**. The results are shown as the density mean ± SEM for each group (*n* = 6). ***P < 0.001.

Toll-like receptors (TLRs) are pattern recognition receptors expressed in a variety of immune cells. Myd88 is the adaptor protein of TLRs that acts as a bridge connecting downstream inflammation signals. To investigate whether r*Sj*-Cys protects atherosclerotic renal damage by inhibiting the expression of TLR2 and Myd88, we detected the expression level of TLR2 and Myd88 proteins in the renal tissues (Figure 10A). The results showed that the expression of TLR2 and Myd88 was remarkably increased in the renal tissues of mice fed with HFD compared with the kidneys in NCD group and the NCD group with r*Sj*-Cys. Treatment with r*Sj*-Cys significantly inhibited their expression in the HFD group. There was no effect of the r*Sj*-Cys itself on the expression of TLR2 and MyD88 in NCD mice (Figure 10B). The results indicate the inhibition of inflammation by r*Sj*-Cys may act through inhibiting the TLR2 and Myd88 signal pathway.

### The Effect of r*Sj*-Cys Induces the Generation of Tregs in the Spleen

**FIGURE 11:**
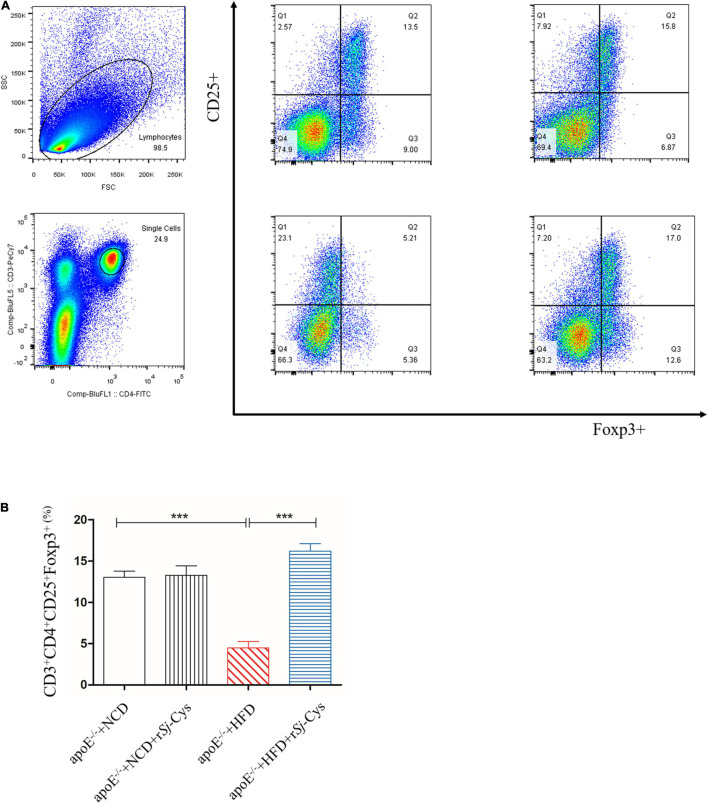
**(A)** Representative data from the FACS analysis of the CD3^+^CD4^+^CD25^+^Foxp3^+^ Tregs and the numbers in the upper right quadrants indicate the positive percentages of Tregs. **(B)** The corresponding bar graphs of the percentage of CD3^+^CD4^+^CD25^+^Foxp3^+^ Tregs. The results are shown as the mean ± SEM for each group (*n* = 6). ***P < 0.001.

To explore whether r*Sj*-Cys reduces the development of atherosclerosis by stimulating Treg, we detected the Treg-expressed CD3ε, CD4, and CD25 on the surface and the intracellular expression of Foxp3 in splenocytes (Figure 11A). The results showed that the rate of CD3ε^+^CD4^+^CD25^+^Foxp3^+^ Treg in the HFD group was significantly lower than that in the NCD group. Treatment with r*Sj*-Cys significantly recovered and boosted the level of CD3ε^+^CD4^+^CD25^+^Foxp3^+^ Tregs. There were no statistical differences between group NCD and group NCD with r*Sj*-Cys (Figure 11B). The results are consistent with the down-regulated proinflammatory cytokines and up-regulted anti-inflammatory cytokines IL-10 and TGF-β in sera and renal tissues measured previously. Our data indicate that the r*Sj*-Cys–stimulated Treg is closely related to the attenuated atherosclerosis.

## Discussion

Apolipoprotein E knockout mice (apoE^–/–^) display poor lipoprotein clearance with subsequent accumulation of cholesterol, leading to atherosclerotic lesions under the feed of HFD (Zhang et al., 1994). In this study, we successfully developed the atherosclerotic lesions in the aorta and aortic sinus of apoE^–/–^ mice after being fed with HFD for 12 weeks, showing the significant lipid deposition and plaque stained by oil red O (Figures 3A–C). The mice with induced atherosclerosis also showed significantly reduced heart function characterized by the increased peak velocity and mean gradient of the ascending aorta (Figure 4) and impaired kidney function with significantly increased levels of Cr and BUN in the sera and the total protein in urine (Figures 5B,C), indicating serious damage of cardiac and renal functions caused by atherosclerosis in the apoE^–/–^ mice fed with HFD. Strikingly, the atherosclerotic lesions were reduced, and the functions of the heart and kidney improved significantly after being treated with r*Sj*-Cys compared to mice without treatment, indicating that r*Sj*-Cys possesses potential as a therapeutic agent for atherosclerosis.

Atherosclerosis is the progressive damage of arteries, which can be caused by various factors such as abnormal blood lipid metabolism and systemic chronic inflammation (Raggi et al., 2018; Shah, 2019). In this study, we demonstrated that the body weight, TC, TG, and low-density lipoprotein cholesterol (LDL-c) were significantly increased, whereas high-density lipoprotein cholesterol (HDL-c) decreased in apoE^–/–^ mice fed with HFD, indicating the imbalanced lipid metabolism (Figure 5A). The diet with high cholesterol and saturated fatty acids can significantly build up body weight, elevate plasma cholesterol levels, and increase the risk of atherosclerosis (Manzini et al., 2019). After being treated with r*Sj*-Cys for 4 weeks, body weight gain stopped in mice, and the mice started to lose weight afterward until the end of the experiment (12 weeks). It is the first time we observed that treatment with r*Sj*-Cys significantly reduced the body weight of mice fed with HFD compared to the mice without r*Sj*-Cys treatment (Figure 2). We also observed that the levels of blood lipids including TC, TG, and LDL-c were reduced, and HDL-c increased significantly after r*Sj*-Cys treatment. The results indicate that r*Sj*-Cys can significantly improve lipid metabolism in apoE^–/–^ mice with HFD. It has long been noticed that helminthic infection was associated with the considerably lower prevalence of obesity (Okada et al., 2013). Mice infected with *S. mansoni* reduced body weight by improving insulin sensitivity and glucose intolerance (Hussaarts et al., 2015). *S. japonicum* infection improved lipid metabolism in mice fed with an HFD, possibly through a soluble worm egg protein called Sjp40 to inhibit host microRNA miRNA-802, resulting in the attenuation of lipogenesis (Ni et al., 2021). Our results are consistent with these observations and further support the negative correlation between helminthic infection or its derivatives and the incidence of obesity and lipid metabolism disorder (Wiria et al., 2012); however, it is still needed to further study how *Sj*-Cys improves the lipid metabolism and reduces the body weight.

As we have already known, atherosclerosis is a chronic inflammatory disease, and macrophages are the major inflammatory cells involved in the pathological development of atherosclerosis (De Paoli et al., 2014; Zhang et al., 2018). Some risk factors such as dyslipidemia, oxidized lipids, and cytokines can activate polarization of M1 macrophages that secrete proinflammatory cytokines such as TNF-α, IL-6, IL-1β, and iNOS, thereby creating inflammatory environment and promoting the development of atherosclerosis (Khallou-Laschet et al., 2010; Colin et al., 2014; Liu et al., 2014; Raggi et al., 2018). In order to evaluate whether r*Sj*-Cys can alleviate the development of atherosclerosis by regulating the polarization and functions of macrophages, the peritoneal macrophages were isolated from mice and stimulated with oxidized LDL in the presence of r*Sj*-Cys. We found that the macrophages were strongly stimulated by the oxidized LDL to release proinflammatory cytokines TNF-α, IL-6, and iNOS. IL-6 has been identified as a major cytokine that promotes atherosclerosis in apoE^–/–^ mice (Fan et al., 2020). Incubation with r*Sj*-Cys significantly suppressed the secretion of these proinflammatory cytokines stimulated by oxidized LDL and simultaneously up-regulated the secretion of IL-10, TGF-β, and Arg-1, the regulatory cytokine/chemokine mostly secreted by M2 macrophages (Figure 8). Our results confirmed oxidized lipid as a strong stimulator for inflammatory macrophages (M1) and the link between lipid oxidation and the macrophage polarization (Adamson and Leitinger, 2011; Xu et al., 2019). This link can be interrupted by r*Sj*-Cys that induces the polarization of macrophages from M1 to M2 and stimulates the secretion of related regulatory cytokines to alleviate the inflammatory responses caused by lipid disorders. As we know, M2 macrophages possess antiatherosclerotic functions by producing high levels of anti-inflammatory cytokines IL-10 and TGF-β to rebuild the cytokine balance to counteract the inflammatory response maintained by M1 macrophages (Colin et al., 2014; Liu et al., 2014). The regulatory effects of r*Sj*-Cys on programming monocytes/macrophages to an anti-inflammatory phenotype have been further confirmed by measuring the proinflammatory and anti-inflammatory cytokines in sera of treated mice. We found that r*Sj*-Cys significantly reduced the levels of TNF-α, IL-6, and iNOS and increased the levels of IL-10, TGF-β, and Arg-1 in sera (Figure 9A). It may provide a novel and feasible approach for the treatment of atherosclerosis by promoting macrophage polarization from M1 to M2 phenotype.

Kidney is one of the major organs affected by atherosclerosis. The renal damage and function failure is the major consequence of atherosclerosis. Hyperlipidemia can lead to atherosclerosis and abnormal lipoprotein deposits in renal tissue damages glomerulus and endothelial cells and induces inflammation in the kidney (Ruan et al., 2009; Bobulescu, 2010). Patients with existing basic renal disease can increase the mortality by accelerating the process of atherosclerosis (Swaminathan and Shah, 2011; Shoji et al., 2012). In this study, we observed not only the significant increase of kidney weight but also the significant deposit of fat and the inflammatory cell infiltration in glomerular tissue of mice fed with HFD (Figures 3D,E). More specifically, we identified that CD86^+^ M1 macrophages (Figure 7) and M1 macrophage–produced IL-6, TNF-α, and iNOS mRNA expression (Figure 9B) were significantly increased in the kidneys of mice fed with HFD, indicating that M1 macrophages and their inflammatory activities are significantly activated in kidneys with atherosclerosis. These results indicate that renal tissue is dominated by inflammatory macrophages when atherosclerotic lesion occurs. In addition, oxidative stress has been shown to play an important role in the development of atherosclerosis (Jiang et al., 2011). Lipid peroxidation is the major risk factor for atherosclerosis, which induces M1 macrophage polarization and promotes inflammatory responses. In this study, we found that MDA, as a marker for oxidative stress, was significantly accumulated in kidneys of HFD-fed mice (Figure 6), further indicating the inflammatory status of kidney during atherosclerosis. The HFD-induced atherosclerosis and inflammation damaged the function of kidney reflected by the increased Cr and BUN levels in sera and increased protein content in urine (Figures 5B,C). Treatment with r*Sj*-Cys not only significantly reduced the kidney weight at the rate even higher than body weight (Figure 2B) but also improved the kidney function associated with reduced fatty acid deposition and less inflammatory cell infiltration and recovered structure of glomerular tissue as well in mice fed with HFD (Figures 3D,E). We further identified that r*Sj*-Cys was able to induce macrophage polarization from M1 (CD86^+^) to M2 (CD206^+^) in renal tissue associated with reduced proinflammatory cytokines (IL-6, TNF-α) and iNOS and increased regulatory or anti-inflammatory cytokines (IL-10, TGF-β) and Arg-1. All results suggest that the therapeutic effect of r*Sj*-Cys on atherosclerotic renal damage takes place through inhibiting renal inflammation by promoting M1 to M2 macrophage polarization.

In recent years, more and more studies have identified that Treg plays a vital role in regulating the progression of atherosclerosis. Decreasing the number of CD4^+^CD25^+^Foxp3^+^ Tregs was related to the progression of atherosclerosis (Butcher et al., 2016; Tian et al., 2017, 2018; Ou et al., 2018). Therefore, increasing the numbers and improving the immune regulation function of Treg may serve as a basic immunotherapy for the treatment of atherosclerosis (Ou et al., 2018). CD4^+^CD25^+^Foxp3^+^ Treg exerts immunomodulatory effects by releasing anti-inflammatory cytokines IL-10 and TGF-β to inhibit the development of atherosclerosis possibly through promoting the conversion of M1 to M2 macrophages (Tiemessen et al., 2007; Lin et al., 2010). As a return, IL-10 and TGF-β also enhance the production and function of Tregs (Ji et al., 2017). In this study, we observed the significant induction of IL-10 and TGF-β in both local renal tissues and in sera of mice treated with r*Sj*-Cys. Further investigation found that CD4^+^CD25^+^Foxp3^+^ Tregs were significantly decreased in HFD-induced atherosclerosis, and the treatment with r*Sj*-Cys significantly stimulated the proliferation of Tregs in splenocytes (Figures 11A,B). The results are consistent with the findings by Chen et al. (2017) that infection of *S. japonicum* stimulated the differentiation of Tregs and our previous findings of the therapeutic effects of r*Sj*-Cys on sepsis related to the increased levels of IL-10 and TGF-β (Li et al., 2017; Xie et al., 2021). Our results strongly suggest that r*Sj*-Cys plays a role in inhibiting inflammation and prevention of atherosclerosis mainly through stimulating T regulatory response. The r*Sj*-Cys itself had little effect on the cytokine profile, suggesting that r*Sj*-Cys mainly exhibits immunomodulatory effects when inflammation occurs.

TLRs are expressed in a variety of immune cells such as monocytes/macrophages and dendritic cells, which can recognize pathogen-associated molecular pattern molecules. TLRs are key players in the pathogenesis of inflammatory diseases (Roshan et al., 2016). Many studies have shown that patients with atherosclerosis increased the expression of TLR2 in atherosclerotic aorta (Chen et al., 2016). The TLR-mediated cascade immune response requires the participation of Myd88 as an adaptor or bridge to connect downstream inflammatory signals, leading to the activation of nuclear transcription factor NF-κB to regulate the expression of inflammatory genes such as TNF-α, IL-1β, and IL-6 (Kawai and Akira, 2007; Gay et al., 2014; Li et al., 2020). TLR2/Myd88 signaling pathway plays an important role in the pathogenesis of atherosclerosis. Blocking the expression of TLR2 or Myd88 reduced the plaque size, lipid level, macrophage infiltration, and proinflammatory cytokine levels in apoE^–/–^ mice (Björkbacka et al., 2004; Wang et al., 2013). In order to investigate whether r*Sj*-Cys exerts antiatherosclerotic effects by inhibiting the TLR2/Myd88-dependent signaling pathway, we measured the expression of TLR2 and Myd88 in kidney tissue of apoE^–/–^ mice. Our study found that the expression of TLR2 and Myd88 was increased in apoE^–/–^ mice fed with HFD. Treatment with r*Sj*-Cys significantly reduced the expression levels of TLR2 and Myd88 in the renal tissues of mice fed with HFD (Figures 10A,B), indicating that the anti-inflammatory effect of r*Sj*-Cys may act through inhibiting the TLR2/Myd88 pathway. However, helminthic infections or their derivatives may exert their effect on antimetabolic diseases through other different mechanisms, such as activating the STAT6 signaling pathway, changing the intestinal microbiota (Yang et al., 2013; Crowe et al., 2020). Any other mechanism involved in the therapeutic effect of r*Sj*-Cys on atherosclerosis and atherosclerotic renal damage needs to be further investigated. Because of the inhibitory activity of r*Sj*-Cys on cathepsins (Vray et al., 2002), it cannot be excluded that r*Sj*-Cys plays its role in lipid metabolism and atherosclerosis through inhibiting cathepsins related to lipid metabolism and atherosclerosis. The imbalance between cystatins and cathepsins has been identified to be involved in the development of atherosclerosis (Wu et al., 2018).

In this study, we revealed that inflammatory macrophages play an important role in the pathogenesis of atherosclerosis. Treatment with r*Sj*-Cys significantly inhibited atherosclerosis and improved kidney functions through promoting macrophage polarization from M1 to M2, therefore inhibiting M1 macrophage–induced inflammation. The possible mechanism underlying the regulatory effect of r*Sj*-Cys on reducing atherosclerosis and atherosclerotic renal damage is that r*Sj*-Cys stimulates Tregs and M2 macrophage polarization to produce regulatory cytokines IL-10 and TGF-β, thereby inhibiting the production of proinflammatory cytokines through inhibiting the activation of TLR2/MyD88 signaling pathway. However, immune regulation of inflammation and atherosclerosis is a complicated process with different mechanisms, except for the macrophage polarization and Treg regulation. For example, more evidence demonstrated that neutrophils act as an important regulator in the atherosclerosis and cardiovascular inflammation (Silvestre-Roig et al., 2020). At the early stage, hypercholesterolemia and hyperglycemia stimulated the production of neutrophils in the bone marrow (Drechsler et al., 2010); the increased neutrophils secreted chemotactic proteins to recruit monocytes, thereby accelerating atherosclerosis and cardiovascular inflammation (Soehnlein et al., 2009). Neutrophils also play roles in accelerating all stages of atherosclerosis by fostering monocyte recruitment and macrophage activation and through cytotoxicity (Silvestre-Roig et al., 2020). It has been known that platelets are also involved in the development and manifestation of atherosclerosis by secreting chemokines including CXCL4 or platelet factor 4, CCL5, CXCL12 to recruit monocytes or neutrophils to promote local inflammatory processes at sites of vascular injury (Bakogiannis et al., 2019). Recent studies also showed that gut microbiota and their metabolites play roles in atherosclerosis (Duttaroy, 2021). Further investigation is needed to determine whether r*Sj*-Cys has an effect on the modulation of neutrophil and platelet functions, or altering gut microbiota, in the process of atherosclerosis. The results in this study provide consolidate evidence that *Schistosoma*-derived cystatin exhibits anti-inflammation property and could be developed as a therapeutic agent to treat lipid metabolism disorder and atherosclerosis that threats million lives around the world.

## Conclusion

This study identified that r*Sj*-Cys stimulated Treg and M2 macrophages to secrete regulatory cytokines IL-10 and TGF-β that inhibit oxidized lipid-induced inflammation and atherosclerosis through inhibiting the TLR2/Myd88 pathway. Therefore, r*Sj*-Cys can be used as a potential therapeutic agent for the prevention and treatment of atherosclerosis.

## Data Availability Statement

The original contributions presented in the study are included in the article/supplementary material, further inquiries can be directed to the corresponding authors.

## Ethics Statement

The animal study was reviewed and approved by Animal Care and Use Committee of Bengbu Medical College (approval no: LAEC-2014-039).

## Author Contributions

XY, HuL, HY, and HoL conceived and designed the study. HY, HoL, ZM, YX, YS, DL, and HG performed the experiments. HY, HoL, WC, YY, XW, and LC analyzed the data. HY wrote the manuscript. BZ, XY, and HuL critically revised the manuscript. All authors contributed to the article and approved the submitted version.

## Conflict of Interest

The authors declare that the research was conducted in the absence of any commercial or financial relationships that could be construed as a potential conflict of interest.

## Publisher’s Note

All claims expressed in this article are solely those of the authors and do not necessarily represent those of their affiliated organizations, or those of the publisher, the editors and the reviewers. Any product that may be evaluated in this article, or claim that may be made by its manufacturer, is not guaranteed or endorsed by the publisher.

## References

[B1] Adamson S. Leitinger N. (2011). Phenotypic modulation of macrophages in response to plaque lipids. *Curr. Opin. Lipidol.* 22 335–342. 10.1097/MOL.0b013e32834a97e4 21841486 PMC3979355

[B2] Aprahamian T. R. Zhong X. Amir S. Binder C. J. Chiang L. K. Al-Riyami L. (2015). The immunomodulatory parasitic worm product ES-62 reduces lupus-associated accelerated atherosclerosis in a mouse model. *Int. J. Parasitol.* 45 203–207. 10.1016/j.ijpara.2014.12.006 25666929 PMC4355381

[B3] Bakogiannis C. Sachse M. Stamatelopoulos K. Stellos K. (2019). Platelet-derived chemokines in inflammation and atherosclerosis. *Cytokine* 122:154157. 10.1016/j.cyto.2017.09.013 29198385

[B4] Barrett T. J. (2020). Macrophages in atherosclerosis regression. *Arterioscler. Thromb. Vasc. Biol.* 40 20–33. 10.1161/ATVBAHA.119.312802 31722535 PMC6946104

[B5] Binder C. J. Chang M. K. Shaw P. X. Miller Y. I. Hartvigsen K. Dewan A. (2002). Innate and acquired immunity in atherogenesis. *Nat. Med.* 8 1218–1226. 10.1038/nm1102-1218 12411948

[B6] Björkbacka H. Kunjathoor V. V. Moore K. J. Koehn S. Ordija C. M. Lee M. A. (2004). Reduced atherosclerosis in MyD88-null mice links elevated serum cholesterol levels to activation of innate immunity signaling pathways. *Nat. Med.* 10 416–421. 10.1038/nm1008 15034566

[B7] Bobulescu I. A. (2010). Renal lipid metabolism and lipotoxicity. *Curr. Opin. Nephrol. Hypertens* 19 393–402. 10.1097/MNH.0b013e32833aa4ac 20489613 PMC3080272

[B8] Bogdan C. Vodovotz Y. Nathan C. (1991). Macrophage deactivation by interleukin 10. *J. Exp. Med.* 174 1549–1555. 10.1084/jem.174.6.1549 1744584 PMC2119047

[B9] Butcher M. J. Filipowicz A. R. Waseem T. C. McGary C. M. Crow K. J. Magilnick N. (2016). Atherosclerosis-driven treg plasticity results in formation of a dysfunctional subset of plastic IFNγ+ Th1/Tregs. *Circ. Res.* 119 1190–1203. 10.1161/CIRCRESAHA.116.309764 27635087 PMC5242312

[B10] Chen L. He B. Hou W. He L. (2017). Cysteine protease inhibitor of *Schistosoma japonicum* - a parasite - derived negative immunoregulatory factor. *Parasitol. Res.* 116 901–908. 10.1007/s00436-016-5363-0 28066871

[B11] Chen X. Cui R. Li R. Lin H. Huang Z. Lin L. (2016). Development of pristane induced mice model for lupus with atherosclerosis and analysis of TLR expression. *Clin. Exp. Rheumatol.* 34 600–608. 27385322

[B12] Cheng Y. Zhu X. Wang X. Zhuang Q. Huyan X. Sun X. (2018). *Trichinella spiralis* infection mitigates collagen-induced arthritis via programmed death 1-mediated immunomodulation. *Front. Immunol.* 9:1566. 10.3389/fimmu.2018.01566 30093899 PMC6070611

[B13] Colin S. Chinetti-Gbaguidi G. Staels B. (2014). Macrophage phenotypes in atherosclerosis. *Immunol. Rev.* 262 153–166. 10.1111/imr.12218 25319333

[B14] Crowe J. Lumb F. E. Doonan J. Broussard M. Tarafdar A. Pineda M. A. (2020). The parasitic worm product ES-62 promotes health- and life-span in a high calorie diet-accelerated mouse model of ageing. *PLoS Pathog.* 16:e1008391. 10.1371/journal.ppat.1008391 32163524 PMC7108737

[B15] De Paoli F. Staels B. Chinetti-Gbaguidi G. (2014). Macrophage phenotypes and their modulation in atherosclerosis. *Circ. J.* 78 1775–1781. 24998279 10.1253/circj.cj-14-0621

[B16] Doenhoff M. J. Stanley R. G. Griffiths K. Jackson C. L. (2002). An anti-atherogenic effect of *Schistosoma mansoni* infections in mice associated with a parasite-induced lowering of blood total cholesterol. *Parasitology* 125(Pt 5) 415–421. 10.1017/s0031182002002275 12458825

[B17] Drechsler M. Megens R. T. van Zandvoort M. Weber C. Soehnlein O. (2010). Hyperlipidemia-triggered neutrophilia promotes early atherosclerosis. *Circulation* 122 1837–1845. 10.1161/CIRCULATIONAHA.110.961714 20956207

[B18] Duttaroy A. K. (2021). Role of gut microbiota and their metabolites on atherosclerosis, hypertension and human blood platelet function: a review. *Nutrients* 13:144. 10.3390/nu13010144 33401598 PMC7824497

[B19] Elliott D. E. Weinstock J. V. (2012). Helminth-host immunological interactions: prevention and control of immune-mediated diseases. *Ann. N. Y. Acad. Sci.* 1247 83–96. 10.1111/j.1749-6632.2011.06292.x 22239614 PMC3744090

[B20] Fan Q. Liu Y. Rao J. Zhang Z. Xiao W. Zhu T. (2020). Anti-atherosclerosis effect of angong niuhuang pill via regulating Th17/treg immune balance and inhibiting chronic inflammatory on ApoE-/- mice model of early and mid-term atherosclerosis. *Front. Pharmacol.* 10:1584. 10.3389/fphar.2019.01584 32082145 PMC7005527

[B21] Förstermann U. Xia N. Li H. (2017). Roles of vascular oxidative stress and nitric oxide in the pathogenesis of atherosclerosis. *Circ. Res.* 120 713–735. 10.1161/CIRCRESAHA.116.309326 28209797

[B22] Gao S. Li H. Xie H. Wu S. Yuan Y. Chu L. (2020). Therapeutic efficacy of *Schistosoma japonicum* cystatin on sepsis-induced cardiomyopathy in a mouse model. *Parasit. Vectors* 13:260. 10.1186/s13071-020-04104-3 32423469 PMC7236195

[B23] Gaweł S. Wardas M. Niedworok E. Wardas P. (2004). Dialdehyd malonowy (MDA) jako wskaźnik procesów peroksydacji lipidów w organizmie [malondialdehyde (MDA) as a lipid peroxidation marker]. *Wiad. Lek.* 57 453–455. 15765761

[B24] Gay N. J. Symmons M. F. Gangloff M. Bryant C. E. (2014). Assembly and localization of toll-like receptor signalling complexes. *Nat. Rev. Immunol.* 14 546–558. 10.1038/nri3713 25060580

[B25] Geovanini G. R. Libby P. (2018). Atherosclerosis and inflammation: overview and updates. *Clin. Sci.* 132 1243–1252. 10.1042/CS20180306 29930142

[B26] Gistera A. Hansson G. K. (2017). The immunology of atherosclerosis. *Nat. Rev. Nephrol.* 13 368–380. 10.1038/nrneph.2017.51 28392564

[B27] Hussaarts L. García-Tardón N. van Beek L. Heemskerk M. M. Haeberlein S. van der Zon G. C. (2015). Chronic helminth infection and helminth-derived egg antigens promote adipose tissue M2 macrophages and improve insulin sensitivity in obese mice. *FASEB J.* 29 3027–3039. 10.1096/fj.14-266239 25852044

[B28] Jiang F. Qian J. Chen S. Zhang W. Liu C. (2011). Ligustrazine improves atherosclerosis in rat via attenuation of oxidative stress. *Pharm. Biol.* 49 856–863. 10.3109/13880209.2010.551776 21554147

[B29] Ji Q. Meng K. Yu K. Huang S. Huang Y. Min X. (2017). Exogenous interleukin 37 ameliorates atherosclerosis via inducing the Treg response in ApoE-deficient mice. *Sci. Rep.* 7:3310. 10.1038/s41598-017-02987-4 28607385 PMC5468328

[B30] Kaplan M. Shur A. Tendler Y. (2018). M1 macrophages but not M2 macrophages are characterized by upregulation of CRP expression via activation of NFκB: a possible role for Ox-LDL in macrophage polarization. *Inflammation* 41 1477–1487. 10.1007/s10753-018-0793-8 29687414

[B31] Kattoor A. J. Pothineni N. V. K. Palagiri D. Mehta J. L. (2017). Oxidative stress in atherosclerosis. *Curr. Atheroscler. Rep.* 19:42. 10.1007/s11883-017-0678-6 28921056

[B32] Kawai T. Akira S. (2007). Signaling to NF-kappaB by toll-like receptors. *Trends Mol. Med.* 13 460–469. 10.1016/j.molmed.2007.09.002 18029230

[B33] Khallou-Laschet J. Varthaman A. Fornasa G. Compain C. Gaston A. T. Clement M. (2010). Macrophage plasticity in experimental atherosclerosis. *PLoS One* 5:e8852. 10.1371/journal.pone.0008852 20111605 PMC2810335

[B34] Li B. Xia Y. Hu B. (2020). Infection and atherosclerosis: TLR-dependent pathways. *Cell Mol. Life Sci.* 77 2751–2769. 10.1007/s00018-020-03453-7 32002588 PMC7223178

[B35] Li D. Zhang L. Dong F. Liu Y. Li N. Li H. (2015). Metabonomic changes associated with atherosclerosis progression for LDLR(-/-) mice. *J. Proteome Res.* 14 2237–2254. 10.1021/acs.jproteome.5b00032 25784267

[B36] Li H. Wang S. Zhan B. He W. Chu L. Qiu D. (2017). Therapeutic effect of *Schistosoma japonicum* cystatin on bacterial sepsis in mice. *Parasit. Vectors* 10:222. 10.1186/s13071-017-2162-0 28482922 PMC5422996

[B37] Libby P. Buring J. E. Badimon L. Hansson G. K. Deanfield J. Bittencourt M. S. (2019). Atherosclerosis. *Nat. Rev. Dis. Primers* 5:56. 10.1038/s41572-019-0106-z 31420554

[B38] Lin J. Li M. Wang Z. He S. Ma X. Li D. (2010). The role of CD4+CD25+ regulatory T cells in macrophage-derived foam-cell formation. *J. Lipid Res.* 51 1208–1217. 10.1194/jlr.D000497 20007839 PMC2853448

[B39] Liu F. Cheng W. Pappoe F. Hu X. Wen H. Luo Q. (2016). *Schistosoma japonicum* cystatin attenuates murine collagen-induced arthritis. *Parasitol. Res.* 115 3795–3806. 10.1007/s00436-016-5140-0 27393379

[B40] Liu M. Chen X. Ma J. Hassan W. Wu H. Ling J. (2017). β-Elemene attenuates atherosclerosis in apolipoprotein E-deficient mice via restoring NO levels and alleviating oxidative stress. *Biomed. Pharmacother.* 95 1789–1798. 10.1016/j.biopha.2017.08.092 28962084

[B41] Liu Y. C. Zou X. B. Chai Y. F. Yao Y. M. (2014). Macrophage polarization in inflammatory diseases. *Int. J. Biol. Sci.* 10 520–529. 10.7150/ijbs.8879 24910531 PMC4046879

[B42] Lu H. S. Kukida M. Daugherty A. (2019). Links lipoproteins to chronic kidney disease and atherosclerosis. *Curr. Opin. Lipidol.* 30 410–411. 10.1097/MOL.0000000000000625 31460944

[B43] Lutgens E. Daemen M. J. (2001). Transforming growth factor-beta: a local or systemic mediator of plaque stability? *Circ. Res.* 89 853–855. 11701610

[B44] Manzini S. Busnelli M. Parolini C. Minoli L. Ossoli A. Brambilla E. (2019). Topiramate protects apoE-deficient mice from kidney damage without affecting plasma lipids. *Pharmacol. Res.* 141 189–200. 10.1016/j.phrs.2018.12.022 30593851

[B45] Moorhead J. F. Chan M. K. El-Nahas M. Varghese Z. (1982). Lipid nephrotoxicity in chronic progressive glomerular and tubulo-interstitial disease. *Lancet* 2 1309–1311. 10.1016/s0140-6736(82)91513-6 6128601

[B46] Ni Y. Xu Z. Li C. Zhu Y. Liu R. Zhang F. (2021). Therapeutic inhibition of miR-802 protects against obesity through AMPK-mediated regulation of hepatic lipid metabolism. *Theranostics* 11 1079–1099. 10.7150/thno.49354 33391522 PMC7738900

[B47] Okada H. Ikeda T. Kajita K. Mori I. Hanamoto T. Fujioka K. (2013). Effect of nematode *Trichinella infection* on glucose tolerance and status of macrophage in obese mice. *Endocr. J.* 60 1241–1249. 10.1507/endocrj.ej13-0312 23985691

[B48] Ou H. X. Guo B. B. Liu Q. Li Y. K. Yang Z. Feng W. J. (2018). Regulatory T cells as a new therapeutic target for atherosclerosis. *Acta Pharmacol. Sin.* 39 1249–1258. 10.1038/aps.2017.140 29323337 PMC6289392

[B49] Radovic I. Gruden-Movsesijan A. Ilic N. Cvetkovic J. Mojsilovic S. Devic M. (2015). Immunomodulatory effects of *Trichinella spiralis*-derived excretory-secretory antigens. *Immunol. Res.* 61 312–325. 10.1007/s12026-015-8626-4 25616617

[B50] Raggi P. Genest J. Giles J. T. Rayner K. J. Dwivedi G. Beanlands R. S. (2018). Role of inflammation in the pathogenesis of atherosclerosis and therapeutic interventions. *Atherosclerosis* 276 98–108. 10.1016/j.atherosclerosis.2018.07.014 30055326

[B51] Robertson A. K. Rudling M. Zhou X. Gorelik L. Flavell R. A. Hansson G. K. (2003). Disruption of TGF-beta signaling in T cells accelerates atherosclerosis. *J. Clin. Invest.* 112 1342–1350. 10.1172/JCI18607 14568988 PMC228445

[B52] Roshan M. H. Tambo A. Pace N. P. (2016). The Role of TLR2, TLR4, and TLR9 in the pathogenesis of atherosclerosis. *Int. J. Inflam.* 2016:1532832. 10.1155/2016/1532832 27795867 PMC5067326

[B53] Ruan X. Z. Varghese Z. Moorhead J. F. (2009). An update on the lipid nephrotoxicity hypothesis. *Nat. Rev. Nephrol.* 5 713–721. 10.1038/nrneph.2009.184 19859071

[B54] Schwartz C. Hams E. Fallon P. G. (2018). Helminth modulation of lung inflammation. *Trends Parasitol.* 34 388–403. 10.1016/j.pt.2017.12.007 29339033

[B55] Shah P. K. (2019). Inflammation, infection and atherosclerosis. *Trends Cardiovasc. Med.* 29 468–472. 10.1016/j.tcm.2019.01.004 30733074

[B56] Shoji T. Abe T. Matsuo H. Egusa G. Yamasaki Y. Kashihara N. (2012). Committee of renal and peripheral arteries, japan atherosclerosis society. chronic kidney disease, dyslipidemia, and atherosclerosis. *J. Atheroscler. Thromb.* 19 299–315. 10.5551/jat.10454 22166970

[B57] Silvestre-Roig C. Braster Q. Ortega-Gomez A. Soehnlein O. (2020). Neutrophils as regulators of cardiovascular inflammation. *Nat. Rev. Cardiol.* 17 327–340. 10.1038/s41569-019-0326-7 31996800

[B58] Smith P. Mangan N. E. Walsh C. M. Fallon R. E. McKenzie A. N. van Rooijen N. (2007). Infection with a helminth parasite prevents experimental colitis via a macrophage-mediated mechanism. *J. Immunol.* 178 4557–4566. 10.4049/jimmunol.178.7.4557 17372014

[B59] Soehnlein O. Lindbom L. Weber C. (2009). Mechanisms underlying neutrophil-mediated monocyte recruitment. *Blood* 114 4613–4623. 10.1182/blood-2009-06-221630 19696199

[B60] Sun S. Li H. Yuan Y. Wang L. He W. Xie H. (2019). Preventive and therapeutic effects of *Trichinella spiralis* adult extracts on allergic inflammation in an experimental asthma mouse model. *Parasit. Vectors* 12:326. 10.1186/s13071-019-3561-1 31253164 PMC6599242

[B61] Swaminathan S. Shah S. V. (2011). Novel inflammatory mechanisms of accelerated atherosclerosis in kidney disease. *Kidney Int.* 80 453–463. 10.1038/ki.2011.178 21697810

[B62] Taleb S. (2016). Inflammation in atherosclerosis. *Arch. Cardiovasc. Dis.* 109 708–715. 10.1016/j.acvd.2016.04.002 27595467

[B63] Tedgui A. Mallat Z. (2001). Anti-inflammatory mechanisms in the vascular wall. *Circ. Res.* 88 877–887. 10.1161/hh0901.090440 11348996

[B64] Tedgui A. Mallat Z. (2006). Cytokines in atherosclerosis: pathogenic and regulatory pathways. *Physiol. Rev.* 86 515–581. 10.1152/physrev.00024.2005 16601268

[B65] Tian Y. Chen T. Wu Y. Yang L. Wang L. Fan X. (2017). Pioglitazone stabilizes atherosclerotic plaque by regulating the Th17/Treg balance in AMPK-dependent mechanisms. *Cardiovasc. Diabetol.* 16:140. 10.1186/s12933-017-0623-6 29084546 PMC5663071

[B66] Tian Y. Liang X. Wu Y. (2018). The alternation of autophagy/apoptosis in CD4+CD25+Foxp3+ Tregs on the developmental stages of atherosclerosis. *Biomed. Pharmacother.* 97 1053–1060. 10.1016/j.biopha.2017.11.013 29136784

[B67] Tiemessen M. M. Jagger A. L. Evans H. G. van Herwijnen M. J. John S. Taams L. S. (2007). CD4+CD25+Foxp3+ regulatory T cells induce alternative activation of human monocytes/macrophages. *Proc. Natl. Acad. Sci. U.S.A.* 104 19446–19451. 10.1073/pnas.0706832104 18042719 PMC2148309

[B68] Torres N. Guevara-Cruz M. Velázquez-Villegas L. A. Tovar A. R. (2015). Nutrition and atherosclerosis. *Arch. Med. Res.* 46 408–426. 10.1016/j.arcmed.2015.05.010 26031780

[B69] van der Vlugt L. E. Labuda L. A. Ozir-Fazalalikhan A. Lievers E. Gloudemans A. K. Liu K. Y. (2012). Schistosomes induce regulatory features in human and mouse CD1d(hi) B cells: inhibition of allergic inflammation by IL-10 and regulatory T cells. *PLoS One* 7:e30883. 10.1371/journal.pone.0030883 22347409 PMC3275567

[B70] van Rooy M. J. Pretorius E. (2014). Obesity, hypertension and hypercholesterolemia as risk factors for atherosclerosis leading to ischemic events. *Curr. Med. Chem.* 21 2121–2129. 10.2174/0929867321666131227162950 24372218

[B71] Vray B. Hartmann S. Hoebeke J. (2002). Immunomodulatory properties of cystatins. *Cell Mol. Life Sci.* 59 1503–1512. 10.1007/s00018-002-8525-4 12440772 PMC11337455

[B72] Wammes L. J. Mpairwe H. Elliott A. M. Yazdanbakhsh M. (2014). Helminth therapy or elimination: epidemiological, immunological, and clinical considerations. *Lancet Infect. Dis.* 14 1150–1162. 10.1016/S1473-3099(14)70771-6 24981042

[B73] Wang S. Xie Y. Yang X. Wang X. Yan K. Zhong Z. (2016). Therapeutic potential of recombinant cystatin from *Schistosoma japonicum* in TNBS-induced experimental colitis of mice. *Parasit. Vectors* 9:6. 10.1186/s13071-015-1288-1 26728323 PMC4700642

[B74] Wang X. X. Lv X. X. Wang J. P. Yan H. M. Wang Z. Y. Liu H. Z. (2013). Blocking TLR2 activity diminishes and stabilizes advanced atherosclerotic lesions in apolipoprotein E-deficient mice. *Acta Pharmacol. Sin.* 34 1025–1035. 10.1038/aps.2013.75 23852085 PMC4003031

[B75] Weinstock J. V. Summers R. W. Elliott D. E. (2005). Role of helminths in regulating mucosal inflammation. *Springer Semin. Immunopathol.* 27 249–271. 10.1007/s00281-005-0209-3 15959781

[B76] Wiria A. E. Djuardi Y. Supali T. Sartono E. Yazdanbakhsh M. (2012). Helminth infection in populations undergoing epidemiological transition: a friend or foe? *Semin. Immunopathol.* 34 889–901. 10.1007/s00281-012-0358-0 23129304

[B77] Witztum J. L. Steinberg D. (1991). Role of oxidized low density lipoprotein in atherogenesis. *J. Clin. Invest.* 88 1785–1792. 10.1172/JCI115499 1752940 PMC295745

[B78] Wolfs I. M. Stöger J. L. Goossens P. Pöttgens C. Gijbels M. J. Wijnands E. (2014). Reprogramming macrophages to an anti-inflammatory phenotype by helminth antigens reduces murine atherosclerosis. *FASEB J.* 28 288–299. 10.1096/fj.13-235911 24043262

[B79] Wu H. Du Q. Dai Q. Ge J. Cheng X. (2018). Cysteine protease cathepsins in atherosclerotic cardiovascular diseases. *J. Atheroscler. Thromb.* 25 111–123. 10.5551/jat.RV17016 28978867 PMC5827079

[B80] Xie H. Wu L. Chen X. Gao S. Li H. Yuan Y. (2021). *Schistosoma japonicum* cystatin alleviates sepsis through activating regulatory macrophages. *Front. Cell Infect. Microbiol.* 11:617461. 10.3389/fcimb.2021.617461 33718268 PMC7943722

[B81] Xu H. Jiang J. Chen W. Li W. Chen Z. (2019). Vascular macrophages in atherosclerosis. *J. Immunol. Res.* 2019:4354786. 10.1155/2019/4354786 31886303 PMC6914912

[B82] Yan K. Wang B. Zhou H. Luo Q. Shen J. Xu Y. (2020). Amelioration of type 1 diabetes by recombinant fructose-1,6-bisphosphate aldolase and cystatin derived from *Schistosoma japonicum* in a murine model. *Parasitol. Res.* 119 203–214. 10.1007/s00436-019-06511-7 31845020

[B83] Yang Z. Grinchuk V. Smith A. Qin B. Bohl J. A. Sun R. (2013). Parasitic nematode-induced modulation of body weight and associated metabolic dysfunction in mouse models of obesity. *Infect. Immun.* 81 1905–1914. 10.1128/IAI.00053-13 23509143 PMC3676010

[B84] Zhang B. Ma Y. Xiang C. (2018). SIRT2 decreases atherosclerotic plaque formation in low-density lipoprotein receptor-deficient mice by modulating macrophage polarization. *Biomed. Pharmacother.* 97 1238–1242. 10.1016/j.biopha.2017.11.061 29145149

[B85] Zhang S. H. Reddick R. L. Burkey B. Maeda N. (1994). Diet-induced atherosclerosis in mice heterozygous and homozygous for apolipoprotein E gene disruption. *J. Clin. Invest.* 94 937–945. 10.1172/JCI117460 8083379 PMC295131

